# Supplementary material for: Sequre: a high-performance framework for secure multiparty computation enables biomedical data sharing
Source: Genome Biol. 2023 Jan 11;24:5. doi: 10.1186/s13059-022-02841-5 (PMC9832703; doi:10.1186/s13059-022-02841-5)
Supplement: Supplementary file 1 — Additional file 1. Sequre supplementary notes provides additional insight to Sequre, its usability and optimizations, and the results [61–90]. [file 13059_2022_2841_MOESM1_ESM.pdf]

Additional file 1: Sequre supplementary notes

Full list of author information is available at the end of the article

1 A short, practical guide to Sequre

We illustrate the usage of Sequre by implementing a secure version of PlassClass [61], a binary classification tool for distinguishing whether a sequence originates from a plasmid sequence or a chromosomal segment. It is based on a binary classification with k-mer counts as feature variables. This example describes how to securely perform the training of the classification model in an MPC environment. Secure evaluation of the trained model on private data for inference can be achieved in a similar manner.

1.1 Data pre-processing and secret sharing

Each data owner prepares the private data to be securely shared with the computing parties. PlassClass begins by counting the number of k-mers (for a predefined k) over a given set of chromosomal and plasmid sequences. These counts will be used as the classifier features. Each data owner can execute this step locally, as it does not require any coordination with other parties. Because Sequre inherits all the functionalities of the Seq language for high-performance bioinformatics pipelines [20], we can use such functionality to quickly implement the k-mer counting in only 10 lines of code as follows:

Code 1 PlassClass preprocessing

```
features = zeros(len(labels), 2 ** 10).to_int()  # k-mer length is 5

def update(label_idx, kmer):
  features[label_idx][int(min(kmer, ~kmer).as_int())] += 1

for fasta_path, label in zip(fastas, labels):
  for seq in seqs(FASTA(fasta_path, fai=False)):
    for kmer in seq.kmers[Kmer[5]](1):
      update(label, kmer)
print("Data preprocessing done!")
```

Note that the original implementation of PlassClass required more than 150 lines of Python code.

Once the classifier features are constructed, we can proceed with *secret sharing*—the process of dividing the private data among the untrusted computing parties without disclosing any private information to each party. We do so by calling Sequre's secret sharing routine:

Code 2 Secret sharing

```
from sequre import secret_share
secret_share(features, labels)
```

Sequre's secret sharing protocol defaults to additive secret sharing [7], which means that one needs to add all shares together to reveal the underlying data. Each data owner executes the secret sharing routine locally to construct the shares. The constructed shares are then distributed to the computing parties via secure channels.

Finally, each client compiles and runs the secret sharing procedure as follows:

Code 3 Client compile and run instruction

```
$ sequre client.seq

Setting up Sequre ...
Compiling client.seq ...
Field size: 170141183460469231731687303715884105727
Ring size: 170141183460469231731687303715884105728

Data preprocessing done!
Connected at 127.0.0.1:9090!
Connected at 127.0.0.1:9091!
Connected at 127.0.0.1:9092!
Secret sharing done!
```

1.2 Configuring the network

Sequre defaults to a `localhost` address. However, each owner (or a *client*) can reconfigure network addresses for the MPC computing parties and the MPC trusted dealer within `dsl/settings.seq` file in Sequre's main directory. Note that this step requires all data holders to agree on the same configuration—Sequre terminates the execution if a mismatch is found between the configurations.

Also, we will assume that the servers used by computing parties are properly deployed and configured (the specifics of deployment are discussed at the end of this section). The following code listing shows an example configuration for a local network of two computing parties running on the same machine:

Code 4 `dsl/settings.seq`—Network configuration

```
# IPs
TRUSTED_DEALER = '127.0.0.1'  # Trusted dealer / Auxiliary party
COMPUTING_PARTIES = [
  '127.0.0.1',  # 1st computing party
  '127.0.0.1'   # 2nd computing party
]
```

1.3 Secure training

Once the data is secretly shared, we can initialize secure training. We will use a linear support vector machine (SVM) classifier for binary classification of sequences. For simplicity, we will optimize regularized hinge loss via the stochastic gradient descent algorithm for our linear SVM. The secure implementation of this procedure in Sequre does not differ much from the straightforward linear SVM pseudocode (see Section 4 for details):

Code 5 Linear SVM training

```
from sequre import dot, zeros_like

mpc, (features, labels) = pool_shares()

@sequre
def lsvm(mpc, X, Y, eta, epochs, l2):
  w = zeros_like(X[0]) + 1
  b = zeros_like(Y[0]) + 1

  for i in range(epochs):
    for feature_vector, label in zip(X, Y):
      z = dot(mpc, feature_vector, w) - b
      # Backward pass
      grad_b = label * ((1 - z * label) > 0)
      grad_w = w * l2 * 2 - feature_vector * grad_b
      w = w - grad_w * eta
      b = b - grad_b * eta

  return w, b
```

In the above code, note how the `lsvm` procedure is decorated by a `@sequre` decorator. This decorator signals to the compiler that the code within the function needs to be executed in an MPC environment. Specifically, it needs to be executed using a fixed set of protocols provided by the `mpc` instance (the first argument of the `lsvm` procedure) that contains the instantiated MPC environment. This environment uses the same settings that data holders used during the secure sharing.

The training procedure can be used as follows:

Code 6 PlassClass training in Sequre

```
1  from sequre import pool_shares
2  mpc, (features, labels) = pool_shares()
3
4  print("Training the linear SVM.")
5
6  weights = lsvm(
7    mpc, features, labels.flatten(),
8    eta=0.01, epochs=10, l2=0.01
9  )
10 print(f"First 10 weights at CP{mpc.pid}: {weights[:10].print(mpc)}")
11 mpc.done()
```

This code will gather the secretly shared data, instantiate the MPC environment (line 2), train a binary classification model on the private data (line 6), reveal the trained weights to the end-users (line 10), and finally notify the data holders and the end-users that the training is complete (line 11).

Note that the above binary classification routine is actually a part of Sequre's standard library and can be easily used in any pipeline through a simple import statement.

1.4 Complete MPC implementation of PlassClass

Here are the final 42 lines of code for our secure MPC version of PlassClass: 15 lines of code for the secret sharing (`client.seq`) program and 27 lines for the training (`server.seq`). Note that the same pipeline would be hundreds of lines of code long if MPC-related optimizations had to be done by hand, even if the multiplication and comparison procedures were provided by Sequre's standard library. Finally,

note the original non-secure PlassClass implementation includes over 300 lines of code in Python.

Code 7 `client.seq`

```
from bio import seqs, Kmer, FASTA
from sequre import secret_share, zeros

features = zeros(len(labels), 2 ** 10).to_int()  # k-mer length is 5

def update(label_idx, kmer):
  features[label_idx][int(min(kmer, ~kmer).as_int())] += 1

for fasta_path, label in zip(fastas, labels):
  for seq in seqs(FASTA(fasta_path, fai=False)):
    for kmer in seq.kmers[Kmer[5]](1):
      update(label, kmer)
print("Data preprocessing done!")

secret_share(features, [labels])
```

Code 8 `server.seq`

```
from sequre import sequre, pool_shares
from sequre import dot, zeros_like

mpc, (features, labels) = pool_shares()

@sequre
def lsvm(mpc, X, Y, eta, epochs, l2):
  w = zeros_like(X[0]) + 1
  b = zeros_like(Y[0]) + 1

  for i in range(epochs):
    for feature_vector, label in zip(X, Y):
      z = dot(mpc, feature_vector, w) - b
      # Backward pass
      grad_b = label * ((1 - z * label) > 0)
      grad_w = w * l2 * 2 - feature_vector * grad_b
      w = w - grad_w * eta
      b = b - grad_b * eta

  return w, b

print("Training the linear SVM.")

weights, bias = lsvm(mpc, features, labels.flatten(),
                     eta=0.01, epochs=10, l2=0.01)
print(f"First 10 weights at CP{mpc.pid}: {weights[:10].print(mpc)}")
mpc.done()
```

1.5 Deployment

So far, we have covered the client's perspective of deploying the secure pipeline (i.e., configuring the network, secret sharing and pipeline execution).

Currently Sequre assumes that the participating computing parties are deployed and accessible on the network. The secure pipeline (`server.seq` in our example) needs to be sent to the computing parties for execution after the code review is completed (expected to be performed by the data owner or a third-party responsible for ensuring security compliance). Users can choose between compiling the source

code privately ahead-of-time and deploying the executable(s) to the servers, or deploying the source code to the servers and using just-in-time compilation for execution. We follow the latter approach in the example bellow. The exact method of deploying the code to the servers is currently left to the users.

Once the code is deployed, each computing party independently executes it. Upon detecting the secure codeblocks (e.g., `import sequre` or a `@sequre` decorator), the compiler will prepend the necessary MPC setup procedures to the codebase. This will allow servers to set up secure communication channels between each other as well as the pseudo-random generators needed for reducing the network bandwidth upon the execution. Finally, after the environment setup, secure computing will commence. The output on each computing node should roughly resemble the following output:

Code 9 Plassclass training—output of the first computing party

```
$ sequre server.seq 1

Setting up Sequre ...
Compiling server.seq ...
Field size: 170141183460469231731687303715884105727
Ring size: 170141183460469231731687303715884105728

Connected at 127.0.0.1:9001
Listening at 0.0.0.0:9003
Listening at 0.0.0.0:9091
Initialized MPC at CP1

Training the linear SVM.
Epoch: 1/10
Epoch: 2/10
Epoch: 3/10
Epoch: 4/10
Epoch: 5/10
Epoch: 6/10
Epoch: 7/10
Epoch: 8/10
Epoch: 9/10
Epoch: 10/10
First 10 weights at CP1:
    [-7.62409, 5.62368, -1.00295, 0.983992, 9.47111, 4.6789, 6.42332,
    4.0334, 1.43668, 0.998597]
```

2 Secure multiparty computation primer

Secure multiparty computation (MPC) is a cryptographic framework that enables private computation over private data owned by multiple parties without disclosing the data to each other. There are different types of MPC frameworks depending on the desired security model and the computational paradigm. These frameworks involve coordination among multiple collaborating parties including *data holders*, *end-users* supplying the analysis pipeline, and *computing parties* (CPs) who execute the computation in a secure distributed manner. Note that there may be overlapping roles, e.g. data holders can also be end-users and computing parties.

Sequre adopts an efficient MPC framework based on *additive secret sharing* in the style of SPDZ [62]. Other alternatives include Shamir's polynomial secret sharing and garbled circuits [63, 7]. Each scheme addresses slightly different use cases and

also different security guarantees for some. In the following, we will give a general description of MPC based on Sequre's framework.

The data holders initiate the computation by *secret sharing* the data with the computing parties. Specifically, each data holder splits each of their private data value x into n shares $[x]_1, [x]_2, \dots [x]_n$, where n is the number of computing parties, such that $x = \sum_{i=1}^{n} [x]_i$ (modulo a predetermined modulus). Then the data holder shares each $[x]_i$ with CP_i respectively for $i = \{1, \dots, n\}$. These shares satisfy the key property that each share individually is indistinguishable from a uniformly random number, thus hiding the underlying secret. Here we adopt the notations from Cho et al. [9], where $[x]_i$ denotes the secret share of x accessible only by CP_i (see Section 4). Computing parties will then execute an interactive protocol in which they perform calculations over the respective secret-shared data, also exchanging messages with other parties to facilitate computation over the underlying private data. Subroutines defined for individual operations (e.g. multiplication) provide theoretical guarantees that the messages exchanged during the computation do not reveal any information about any party's private data. Once the computation is complete, the result is revealed by combining the final shares and made available to the end-users.

The above additive secret sharing scheme maintains the two invariants: (i) no computing party can infer any information about the private data that they do not already have access to; and (ii) the shares are constrained to have a sum (modular addition) equal to the secret value they collectively represent. Because of this constrained data representation, performing computation over the private data (which are not directly accessible), e.g. multiplying two secret numbers, requires the use of special operations which typically require the parties to interact and exchange messages (see Section 4 for details). Note that in Sequre, we adopt an efficient server-aided model of MPC, which means that one computing party plays the role of a *trusted dealer* who does not receive any private data (even secret shares) and only generates correlated random numbers in a preprocessing step to be shared with other computing parties in order to facilitate the main protocol. While this weakens the security model by requiring the trusted dealer not to collude with any other party, the resulting performance gain is significant, which Sequre prioritizes.

The security of a MPC framework is typically expressed in terms of the power of the anticipated adversary (e.g. a rogue computing party). Common models include *honest-but-curious* and *malicious* adversaries. An honest-but-curious party will faithfully follow the computational protocol as given, but may attempt to perform additional analysis based on the observed data during the protocol to infer information about the private data. A malicious party is additionally allowed to deviate from the prescribed computational protocol, potentially sending fabricated information in order to extract private information. Based on the type of adversary addressed by the chosen security model, MPC operations need to be revised accordingly to provide adequate protection. In general, stronger schemes that guard against malicious parties tend to be more computationally expensive. Sequre adopts the honest-but-curious security model.

Some MPC frameworks support only a limited number of computing parties (e.g. garbled circuits are for two parties). Frameworks that can be instantiated with more

computing parties arguably offer stronger security since compromising privacy based on secret shared data typically requires compromising all (or the majority of) the computing parties.

Another aspect worth noting is that additive secret sharing-based frameworks can be instantiated with different types of finite algebraic structure for the modular arithmetic required. Two popular choices are a finite field (integers modulo a prime; $\mathbb{Z}_p$) or a ring of integers with a power-of-two modulus ($\mathbb{Z}_{2^k}$). Fractional numbers are commonly represented using fixed-point numbers, incurring some level of precision loss, which can be controlled with the choice of modulus size. Recently, $\mathbb{Z}_{2^k}$ rings are becoming more widely adopted due to their superior performance on modern computer architectures leveraging native integer operations. However, $\mathbb{Z}_{2^k}$ rings support a more limited set of operations as modular inverse generally does not exist, which some operations require. Developing efficient MPC protocols based on different secret sharing schemes and security assumptions is an active area of research.

2.1 Related work

The first comprehensive survey on MPC frameworks [21] measured the expressiveness, accessibility, level of security, and functionality of 11 different frameworks [64, 65, 66, 67, 73, 45, 42, 68, 43, 69, 70] across three simple applications. Today, there are more than 23 available MPC frameworks [64, 65, 66, 67, 46, 45, 42, 68, 43, 69, 70, 38, 39, 40, 41, 44, 19, 71, 72, 73, 74, 75, 76], each offering a different MPC flavour regarding accessibility, security, functionality, or performance.

While the lack of generality, standardization, and reliability is a crucial obstacle to their wide-use adoption [6, 21], the following frameworks come close and are as such closest to Sequre in terms of design and features are MPyC [38] and MP-SPDZ [46]; details and comparisons are available in Section 3.4.

Other related work includes the frameworks evaluated in Section 4 [19, 42, 77]. Lastly, we should mention various applications that use MPC in practice [78, 79, 80], especially those within the field of biomedicine [15, 8, 16, 14, 9, 10, 23, 11, 12, 13].

3 Experiment details

A typical secret-sharing-based MPC pipeline currently consists of three general stages: (i) data holders prepare the data locally and share it with computing parties over secure channels, (ii) computing parties combine the data from data holders and execute the desired pipeline on top of it in a secure manner, and (iii) results are broadcasted back to the end-users and data holders either publicly or privately, where they are reconstructed locally after pooling the outputs from all computing parties. Computation in the first and third stages is local and private, while the second stage is executed in a distributed manner over multiple computing parties.

Sequre adds support for the online distributed computing of a second stage. For local (offline) computing, it internally shifts to Seq [20] for bioinformatics-specific computation and the general-purpose tasks.

Our benchmarks measure the following aspects for each application: (i) *expressiveness* as the number of lines needed for implementation (lines of code, LOC) with all the comments, logging, line breaks, and blank lines removed, (ii) *network*

utilization as the number of bytes exchanged between the computing parties during the execution, and (iii) *runtime* as the number of seconds needed for execution. The offline pre-processing and secret-sharing is excluded from the total runtime in all experiments. Where possible, we also keep track of the network-related and MPC-speficific parameters, such as the number of Beaver partitions (see Section 4.2.3), which measure the effectiveness of our network-related compile-time optimizations. Furthermore, as mentioned in previous chapter, secret-sharing-based MPC paradigms operate within a finite algebraic structure such as Galois field or $\mathbb{Z}_{2^k}$ ring. The size of the algebraic structure directly impacts the performance and security of the deployed protocols as the larger fields or rings increase the security at the cost of the performance (see Section 4.3.5). Sequre defaults to 128-bit long structures.

For each application, we ran Sequre in two modes: one with the optimizations disabled (see Section 4 for their overview), and the other with the optimizations enabled. To ensure fairness, all benchmarks were also conducted in a single-threaded mode and, where possible, coerced to use the same security constraints. Finally, the benchmarks were executed in two network environments: on a single machine, with the network latency reduced to a minimum, and within a local-area network. (Section 5).

3.1 Secure Genome-Wide Association Study (GWAS)

Our implementation of GWAS pipeline is a reimplementation of the existing state-of-the-art Secure-GWAS pipeline [9].

In population stratification analysis, a specific randomized principal component analysis (PCA) is employed for better performance [9]. It utilizes several secure MPC variants of linear algebra routines, such as QR factorization and eigendecomposition—all of which are fully reimplemented in Sequre and individually benchmarked (see Table S1). The specifics on the randomized PCA and the Cochran-Armitage trend test employed in third step are available in Cho et al. [9] (protocols 32 and 33 in the Supplementary Note 9).

We cumulatively benchmarked a set of four linear algebra routines employed in Secure-GWAS: (i) QR factorization, (ii) tridiagonalization, (iii) eigendecomposition, and (iv) orthonormal basis calculation on top of randomly generated matrix $M \in \mathbb{R}^{50\times 50}$. Other costly MPC arithmetic routines, such as secure householder transformation and fixed-point square root and division, are used as the building blocks for the benchmarked routines, and are thus benchmarked implicitly.

Table S1 shows the expressiveness, runtime, and network utilization of Sequre-GWAS and Secure-GWAS (C/C++) implementations across the four methods. The reimplementation of all four methods resulted in 66 lines of Sequre code—6× fewer than the original C/C++ codebase. Further, Sequre did 1.36× fewer Beaver partitions, which resulted in 1.20× reduced network bandwidth and, consequentially, the overall runtime improvement, as Sequre is 1.34× faster than the C/C++ on a Galois field. Note, however, that the further improvements can be made by switching from Galois field to $\mathbb{Z}_{2^k}$ rings. In that case, we achieve 1.6× speedups over the C/C++ version. The majority of performance increases in this case come from the utilization of the more efficient modulus operator (either through our own implementation or an implementation from [81]).

Table S1 Expressiveness, runtime, and network statistics for the original Secure-GWAS (C/C++) implementation (bottom), together with its linear algebra subroutines (top), and their Sequre reimplementations. GWAS was ran on top of a lung cancer dataset reduced to 3,000 individuals and 30,000 SNPs [9]. Sequre is presented with and without the compile-time optimizations enabled (i.e. network, pattern-matching, matrix and modulus optimizations). Runtime is given in the hh:mm:ss format. Bandwith is expressed in megabytes (MB).

			Runtime		Network	
		LOC	On field	On ring	Bandwidth	Beaver partitions
Lin. alg.	C/C++	395	0:00:59	N/A	455	1.31 mil.
	Sequre (w/o opt)	66	0:01:20	0:00:57	499	1.97 mil.
	Sequre (w/ opt)	66	**0:00:44**	**0:00:37**	**378**	**0.96 mil.**
GWAS	C/C++	1,142	3:21:08	N/A	10,254	2.41 mil.
	Sequre (w/o opt)	117	7:09:02	2:26:07	151,309	3.61 mil.
	Sequre (w/ opt)	117	**2:02:22**	**0:53:43**	**9,887**	**2.34 mil.**

Table S2 Runtimes (hh:mm::ss) and speed-up of Sequre over C/C++ baseline measured on top of a lung cancer dataset for different number of individuals and SNPs. Note that the speed-up is preserved as the dataset size increases.

Indiv.	SNPs	Secure-GWAS	Sequre-GWAS	Speed-up
1,000	10,000	0:20:16	0:05:07	3.95×
	20,000	0:40:20	0:10:13	3.93×
	30,000	1:00:42	0:14:54	4.07×
2,000	10,000	0:40:20	0:09:48	4.11×
	20,000	1:14:52	0:19:41	3.80×
3,000	10,000	0:55:49	14:45	3.78×

GWAS was benchmarked on top of a genotype matrix of 3,000 individuals and 30,000 SNPs with the top five principal components being selected in the population stratification analysis and ten covariates added to it before the trend test. In the original codebase, the Beaver partitions were reused manually where possible. Despite that, Sequre version needed 70,225 fewer Beaver partitions than the original codebase. As a result, the total network consumption was reduced by 4%. The overall speedup (3.7×) when compared to the original implementation is primarily due to Sequre's modulus and matrix optimizations (Section 4.3.4), and shifting to $\mathbb{Z}_{2^k}$ ring instead of the Galois field. Also, some portions of the pipeline, such as the Cochran-Armitage test, benefited more than the rest from these optimizations: the network utilization was reduced 28 times, while the runtime was reduced 2× when compared to the non-optimized counterpart. The runtime improvement is projected to be more pronounced in the wide-area network setups because our local test environments had low network latency. Finally, the overall accuracy of Sequre-GWAS is preserved or even marginally improved when compared against the original C++ implementation (Figure S2). More

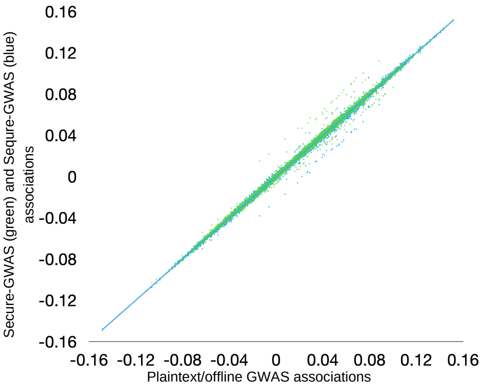

Fig S1 Accuracy comparison of Secure-GWAS (C++; green) and Sequre-GWAS (Sequre; blue) against the offline GWAS implementation (diagonal). The error mean is $\mathbf{9.3 \times 10^{-4}}$ for Secure-GWAS and $\mathbf{8.5 \times 10^{-4}}$ for Sequre-GWAS.

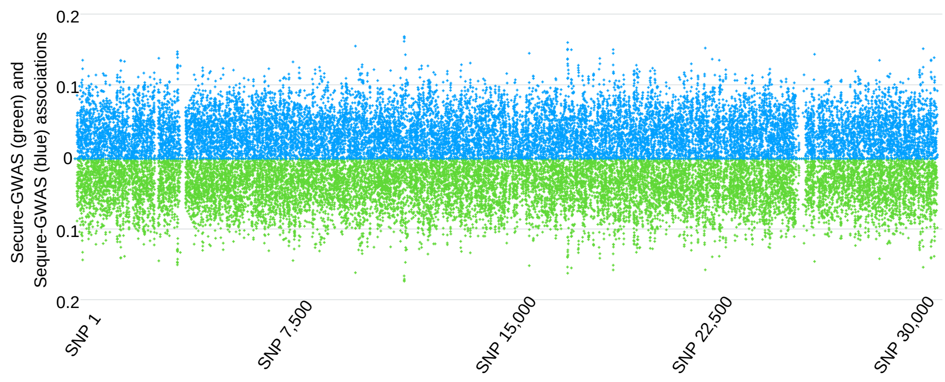

Fig S2 GWAS accuracy comparison between Secure-GWAS (C++; green) and Sequre-GWAS (Sequre; blue) on lung cancer dataset for 30,000 SNPs. Associations accuracy (χ^2 value on y-axis) was preserved in Sequre-GWAS. The two set of results are presented in a mirrored fashion.

precisely, when compared to the offline GWAS implementation (Figure S1), the error mean (i.e., the mean of the difference in accuracy over all points) is 8.5×10^{-4} for Sequre-GWAS—a marginal improvement over the 9.3×10^{-4} that the original C++ implementation achieves. These marginal improvements are observable regardless of the MPC settings used: a Sequre version on rings with 20-bit field precision (theoretically, the least "accurate" mode as compared to the original implementation) achieves better accuracy (8.8×10^{-4}) than the "ideal" 30-bit C++ version on Galois fields (9.2×10^{-4}).

3.2 Secure Drug-Target Interaction (DTI) Prediction

The drug-target interactions (DTI) inference is a binary classification problem that infers whether there is an interaction between a compound and a protein or not for a given set of compounds and proteins.

We used Sequre to reimplement a solution by Hie et al. [10], which employs a neural network for training and inference and uses a binary encoding of the chemical compounds and proteins as features. The simplified molecular-input line-entry system (SMILES) representation of the chemical compounds from STITCH and DrugBank datasets was converted to an extended connectivity fingerprint with diameter 4 (ECFP4) via JChem Base (version 17.28.0, 2017, ChemAxon, `http://www.chemaxon.com`) to obtain a binary feature vector from $\{0, 1\}^{1024}$ for each compound [10]. Similarly, each protein was encoded as a one-hot binary vector corresponding to its Pfam family. There are 5,879 unique Pfam families for the protein identifiers in the STITCH dataset and 1,129 in the DrugBank dataset, yielding the feature vectors of the same respective lengths. The two representations cannot be combined (i.e. one and only one representation is leveraged as a protein feature in our use case). Then, the corresponding feature vectors of each drug-target pair were concatenated and fed into an inference model: a neural network with configurable number of layers and neurons. Rectified linear units (ReLUs) were used as activation functions and a hinge loss was used as a target loss function. Finally, a mini-batch gradient descent was employed over a predefined number of epochs (further details are available in the supplementary notes of Hie et al. [10]).

Our test case used the protein identifiers from the STITCH dataset, resulting in input feature vectors from $\{0,1\}^{6,903}$. It also employed a neural network with one hidden layer of a hundred neurons with a zero dropout and 50 epochs of mini-batch gradient descent. We compared the original C/C++ implementation with our Sequre reimplementation, as well as the SyMPC/PySyft reimplementation of the same pipeline [19]. Table S3 shows the improvements of the Sequre version when compared to the C/C++ baseline and SyMPC/PySyft. As can be observed, the improvement trends and the breakdown of the improvement factors are similar to those observed in Sequre-GWAS in the previous subsection.

Table S3 Expressiveness, runtime, and network statistics for the drug-target inference (DTI) implementations in C/C++, PySyft, and Sequre. Sequre is presented with and without the compile-time optimizations enabled (i.e. network, pattern-matching, matrix and modulus optimizations). Runtime is given in the hh:mm:ss format. Bandwith is expressed in megabytes (MB).

		Runtime		Network		
	LOC	On field	On ring	Bandwidth	Beaver part.	Accuracy
C/C++	274	0:09:18	N/A	3,436	1.04 mil.	0.90 ± 0.05
SyMPC/PySyft	117	N/A	0:04:40	**1,816**	N/A	0.90 ± 0.05
Sequre (w/o opt)	117	0:10:54	0:02:46	2,846	1.04 mil.	0.90 ± 0.05
Sequre (w/ opt)	117	**0:03:59**	**0:02:16**	2,651	**1.04 mil.**	0.90 ± 0.05

We observed that SyMPC/PySyfy has better network utilization than Sequre (Table S3). This is due to the fact that SyMPC uses 64-bit data types for MPC operations as compared to Sequre's default of 128 bits that are twice as large. However, 64-bit data types offer lower statistical security. Also, despite using slower datatypes (both in terms of CPU and network use), Sequre is still nearly two times faster than SyMPC. In terms of accuracy—calculated as the sum of true positive and true negative results divided by the total number of test cases—we observed the same trends in each framework. Depending on the initial random weights, the accuracy in each framework varied from 0.85 to 0.95. Finally, we would like to note that we were unable to run any version of SyMPC or PySyft in a full network (LAN) setup; all versions at the time of writing offer only a proof-of-concept setup that can only be run on a single machine.

3.3 Secure Metagenomic Binning

Metagenomic binning is the classification of the present microbiomes in a sequenced sample. This is different from taxonomic profiling [34], which measures the relative abundance of microbiomes in metagenome sample, and for which a secure solution already exists [82]. We used Sequre to, for the first time, implement secure variants of two existing classification pipelines: Ganon [22] and Opal [23].

Ganon uses k-mer index in the form of an interleaved Bloom filter (IBF) [35] for read classification. For each read, its k-mers are queried against the index, and the read is assigned to the bin with the highest count of matching k-mers. Reads with the insufficient matches are filtered out via the k-mer counting (q-gram) lemma [83]. As the index is public during the inference, index building is done through the original offline algorithm.

An IBF is a matrix whose columns correspond to the Bloom filters for each available bin. That is, the i-th row of an IBF corresponds to the vector of i-th entries of the available Bloom filters. This design allows more efficient joint queries of the

index as follows. For querying a k-mer t, for each available hash function h_i Ganon computes its hash $t_i = h_i(t)$ and obtains the t_i-th row of the IBF matrix. Then it performs an element-wise logical and between the obtained rows to get the final result—the bit-vector in which a non-zero value at position j means that t belongs to a j-th bin in the index. Our secure implementation follows the same procedure with minor differences over the original implementation. First, the IBF is encoded as a list of integers, such that j-th element of the i-th row in the original IBF corresponds to the j-th bit of the i-th integer in the new IBF. The hashes $h_i(t)$ are computed for each k-mer t in offline fashion and then secretly shared between the computing nodes. This enables us to query the encoded IBF directly via an oblivious array getter (Section 4.2) in order to access the secretly shared value of IBF$[h_i(t)]$ for each secretly shared hash value. Finally, to compute the bit-wise logical and between the IBF$[h_i(t)]$, we use the secure bit-decomposition protocol [87]—a part of the Sequre standard library—together with the secure element-wise multiplication of the bit-decomposed values. The result is a secretly shared bit-vector that contains the same information as its offline counterpart, i.e. the list of bins that contain the target k-mer.

We evaluated Sequre-Ganon on the sample dataset from Opal [23] that contains 10 bacterial metagenomes. We built an index from these metagenomes. This dataset also comes with 10,000 microbiome labeled reads of length 65 that can be used for evaluation classification. The size of each Bloom filter in the index was 4,801,571 with the "technical number" of bins equal to 64 (note that the actual number of bins—10 in our case—differs from the technical number; see [22] for details). The k-mer size k was set to 19, and the number of hash functions in the Bloom filter was 4. Expressiveness, runtime, network and accuracy details are provided in Table S4. Note that secret-sharing the input data takes additional 47 hours in a naïve implementation of Ganon that we use at the moment.

The second pipeline—Opal [23]—encodes reads as a features through Gallager encoding [37] and uses support vector machine (SVM) classifier from the Vowpal-Wabbit library [84] for training and classification. Since the exact reimplementation of Vowpal-Wabbit and its various parameters is outside of the scope of this work, and since the performance of different classification algorithms is orthogonal to the problem itself, we instead used a stochastic gradient descent based binary classification with hinge loss and L_2 regularizer—built in 13 lines of code—as a variant of a linear SVM. We also modified Opal to use the same binary classification algorithm for fair comparison. For the sake of completeness, we also provide detailed comparisons with the original Vowpal-Wabbit implementation in Table S4. It is important to note that, unlike Ganon, Opal trains the classification model from the reads themselves, and not from the reference genomes. Thus, different coverage of the training set yields different classification model. Thus we evaluated Sequre-Opal with two training sets: $0.1\times$-deep and $15\times$-deep training sets simulated from the 10 reference metagenomes without error.

Table S4 shows the improvements of Sequre-based implementations over the their non-optimized counterparts. For the reference, the performance and accuracy of the original non-secure implementations is also provided. In case of Sequre-Ganon, the accuracy remains the same as the algorithms are identical between the non-secure

and secure implementation. The accuracy is measured as the percentage of correct matches within the test set. The same holds for Sequre-Opal when compared to the Opal with linear SVM. However, when compared to Opal that uses Vowpal-Wabbit for classification, the accuracy varies and is either $3\times$ better or $1.4\times$ worse, depending on the training depth of coverage. (We also note that adjusting the Vowpal-Wabbit's hyperparameters also produces changes of the same magnitude in the Opal's performance; thus, the question of "the best" model for the purposes of Opal classification needs further study.)

Table S4 Expressiveness, runtime, network and accuracy stats for secure metagenomic binning implementations on top of 10 microbiomes. Opal was evaluated in two offline setups: the one based on Vowpal Wabbit (VWabbit) and the other based on custom Python implementation of linear SVM (PythonSVM). Ganon's offline implementation was done is Seq language. Sequre is presented with and without the compile-time optimizations enabled (i.e. network, pattern-matching, matrix and modulus optimizations). Runtime is given in hh:mm:ss format. Online bandwith is expressed in MB. Beaver partitions are expressed in millions.

		LOC	Runtime (s)	Bandwidth	Beaver part.	Accuracy
Opal[1]	VWabbit	264	0:00:09	N/A	N/A	0.102
	PythonSVM	150	0:00:04	N/A	N/A	0.316
	Sequre (w/o opt)	113	0:01:16	370	2.68	0.316
	Sequre (w/ opt)	113	0:01:08	229	1.93	0.316
Opal[2]	VWabbit	264	0:09:07	N/A	N/A	0.760
	PythonSVM	150	0:09:44	N/A	N/A	0.540
	Sequre (w/o opt)	113	3:36:46	55,035	402.22	0.540
	Sequre (w/ opt)	113	3:07:05	33,941	290.49	0.540
Ganon	Seq-lang	80	0:00:07	N/A	N/A	0.872
	Sequre (w/o opt)	80	42:30:18	96,141	55.88	0.872
	Sequre (w/ opt)	80	18:29:23	81,186	55.72	0.872

[a]Opal with $0.1\times$ coverage simulated reads from 10 microbiomes.
[b]Opal with $15\times$ coverage simulated reads from 10 microbiomes.

3.4 Sequre and other MPC frameworks

We provide a cross-comparison between Sequre and ten mature MPC frameworks included in the recent survey of existing MPC compilers and frameworks [21] (note that, while there are more available frameworks available, most of them are still in the early prototype stage and cannot be successfully run in practice [6, 21]). The following benchmarks were adopted from this survey [21] (Table 3.4) and slightly altered for better scalability:

- `mult3`: a simple multiplication and addition of three numbers;
- `innerprod`: an inner product between two vectors containing 100,000 elements; and
- `xtabs`: a cross table aggregation (joining the two tables by key attributes and computing the sum of the values).

Each evaluated framework offers some novelty concerning the security, expressiveness or performance. Four of these frameworks involved operate in different MPC setups (e.g., garbled circuits) than Sequre, while the other six operate in the same (i.e., an honest-but-curious security model based on additive secret sharing scheme) or less secure MPC setups (e.g., an honest-majority replicated secret sharing with pseudo-random zero sharing) [46, 85]. The secret-sharing type frameworks can operate in different setups, either using 128-bit or 64-bit long integers, or operating within $\mathbb{Z}_p$ field or $\mathbb{Z}_{2^k}$ ring. Thus we evaluated Sequre in the 128-bit setup, where it

was still faster than the 64-bit setups used by the other frameworks (see Table 3.4). We also note that, except for ABY, each framework utilizes pseudo-random generators with shared seeds to reduce the communication rounds between computing parties, just like Sequre. Additionally, Sequre employs a trusted dealer—a non-colluding auxiliary party that generates and distributes correlated randomness via secret sharing to be used in the main protocol (e.g., for the construction of Beaver partitions). We found that other frameworks, excluding Jiff, do not employ such an auxiliary party. However, a similar scheme is employed in Sharemind; it is based on a three-party setting with at most one malicious party, and only 2 out of 3 parties provide private input data for the computation. Furthermore, several recent frameworks (e.g., PySyft, AriaNN), though not included in the benchmark survey reproduced in this analysis, adopt the setting with a trusted dealer for performance; see our DTI prediction results for a comparison between Sequre and PySyft. Here is the overview of the evaluated frameworks and their results:

ABY is a two-party MPC framework embedded in C/C++ with semi-honest computing parties. It provides similar security guarantees as Sequre, and unlike Sequre, it supports garbled circuits and conversion between them and other secret sharing protocols. ABY is considered faster, but also less expressive framework. Compared to Sequre, ABY is up to 6× less expressive on average in the selected benchmarks. It is also slower than Sequre: from 4× slower (`xtabs`) to 1,700× slower (`mult3`).

EMP is a MPC framework based on garbled circuits and embedded in C/C++. Sequre is roughly 6× more expressive on average. It is approximately 21× faster in `mult3` and 276× faster than EMP in `innerprod`. However, EMP is 5.5× faster than Sequre in `xtabs` due to the more advanced implementation of oblivious data structures.

Frigate is a C-like domain-specific language for MPC based on binary circuits. Unfortunately, we were unable to successfully run the benchmarks with the instructions provided by the survey. Nevertheless, Sequre needed on average 4× fewer lines of code for the three benchmarks.

Jiff is a secret sharing and honest-majority-based MPC framework implemented in JavaScript for use in web applications. It required 4× more lines of code than Sequre to implement the three benchmarks. Sequre was also 12× faster in `mult3`. Unfortunately, the code in the survey was obsolete and could not be evaluated for the last two benchmarks (`innerprod` and `xtabs`).

MP-SPDZ is an MPC framework focused on providing support for various MPC variants. It comes with the a custom compiler framework akin to Sequre that includes a novel Python-like domain-specific language. The source code is compiled to bytecode and executed on a custom virtual machine. However, the existing compiler pipeline is still in a proof-of-concept shape. It is implemented in Python, and it employs a set of static compile-time optimizations, such as loop unrolling and software prefetching to reduce network latency. We benchmarked an honest-majority non-malicious replicated secret sharing variant of MPC, optimized via pseudo-random zero secret sharing [86], in MP-SPDZ—one of the fastest variants supported by MP-SPDZ. This variant also leverages the pseudo-random generators to reduce the network communication on secret sharing. Computing

on rings is relatively efficient in MP-SPDZ with the 2.5× speed-up over Sequre in the `xtabs`. However, it is still up to 9× slower than Sequre in the first two (`mult3` and `innerprod`) benchmarks, and 1.4× slower on field in the third benchmark, despite using a weaker and generally faster MPC scheme.

MPyC is a Python library for MPC based on Shamir's secret sharing with semi-honest computing parties. Being a Python library, its syntax and semantic is most similar to that of Sequre; however, it does not posses compile-time optimization capabilities. It has the same code complexity in `mult3` and `xtabs` benchmarks. Still, it required 2× more lines to implement the `innerprod` benchmark. Performance-wise, it was 9× and 14× slower in the `mult3` and `xtabs` benchmark respectively, and up to 250× slower when computing the inner product in the `innerprod` benchmark.

Obliv-C is a garbled-circuits-based MPC with semi-honest computing parties embedded in C. Sequre is up to 1000× faster in the `mult3` and more than 1100× faster in `innerprod` benchmarks. In `xtabs`, despite leveraging a dynamic programming approach (unlike Sequre and other frameworks that use a straightforward brute-force algorithm), it was 2× slower than Sequre (and needed nearly 10× larger codebase for its implementation).

Oblivm is a garbled-circuits-based MPC with semi-honest computing parties embedded in Java. Sequre was significantly faster: from 30× in `xtabs` and 1,600× in `mult3`, to 32,900× in `innerprod`.

Picco is an MPC framework based on Shamir's secret sharing with semi-honest adversaries and implemented in C. While Picco was only 50% less expressive than Sequre, we were unable to run the these benchmarks in practice.

Sharemind is an additive three-party MPC framework based on secret sharing with semi-honest adversaries and an honest majority setup. Sharemind provides its custom domain-specific language for MPC operations. The full version of the framework is not publicly available. However, prototyping the simple MPC programs is enabled through the use of their free SDK (`https://sharemind-sdk.github.io/`). It should be noted that Sharemind, like ABY and Jiff, offers the security model and the MPC variant most similar to that of Sequre. However, unlike Sequre, Sharemind supports only 64-bit $\mathbb{Z}_{2^k}$ rings. Nevertheless, Sequre was faster in all three benchmarks (28× in `mult3`, 1.33× in `innerprod`, and 50× faster in `xtabs`) while maintaining the same level of expressiveness.

3.5 Local-area network environment

So far, all the benchmarks were evaluated in a simulated network environment (with `AF_UNIX` sockets) on a single machine. We also evaluated the four main applications (GWAS, DTI, Opal, and Ganon) in a local-area network environment where the role of each computing party was performed by a different machine.

4 Implementation details

4.1 Notation

The MPC notation in our work is adopted from Cho et al. [9]. While Sequre supports an arbitrary number of computing parties (CPs) and data holders (SPs, also known

Table S5 A cross-comparison between Sequre and ten state-of-the-art MPC frameworks. Frameworks were benchmarked for expressiveness (in terms of lines of code (LOC)) and runtime over multiple MPC setups. Some variants are not supported (marked with ⊥), while some could not be evaluated (marked with N/B) for the provided code samples.

	Framework	LOC	Runtime (ms) 128bit $\mathbb{Z}_p$	128bit $\mathbb{Z}_{2^k}$	64bit $\mathbb{Z}_p$	64bit $\mathbb{Z}_{2^k}$	GC[1]
mult3	ABY	20	⊥	170	⊥	170	⊥
	EMP	25	⊥	⊥	⊥	⊥	**2.1**
	Frigate[2]	19	⊥	⊥	⊥	⊥	N/B
	Jiff	20	⊥	⊥	1.2	⊥	⊥
	MP-SPDZ	**4**	1.0	0.9	0.7	0.6	⊥
	MPyC	8	⊥	⊥	0.9	⊥	⊥
	Obliv-C	11	⊥	⊥	⊥	⊥	100
	Oblivm	10	⊥	⊥	⊥	⊥	160
	Picco[2]	6	N/B	N/B	N/B	N/B	⊥
	Sharemind	**4**	⊥	⊥	⊥	2.8	⊥
	Sequre	**4**	**0.2**	**0.1**	–	–	⊥
innerprod	ABY	30	⊥	520	⊥	900	⊥
	EMP	28	⊥	⊥	⊥	⊥	**4,700**
	Frigate[2]	18	⊥	⊥	⊥	⊥	N/B
	Jiff[2]	20	⊥	⊥	N/B	⊥	⊥
	MP-SPDZ	7	78	45	77	44	⊥
	MPyC	7	⊥	⊥	4,200	⊥	⊥
	Obliv-C	13	⊥	⊥	⊥	⊥	19,000
	Oblivm	21	⊥	⊥	⊥	⊥	560,000
	Picco[2]	6	N/B	N/B	N/B	N/B	⊥
	Sharemind	**4**	⊥	⊥	⊥	20	⊥
	Sequre	**4**	**24**	**17**	–	–	⊥
xtabs	ABY	50	⊥	210	⊥	200	⊥
	EMP	25	⊥	⊥	⊥	⊥	**9**
	Frigate[2]	45	⊥	⊥	⊥	⊥	N/B
	Jiff[2]	25	⊥	⊥	N/B	⊥	⊥
	MP-SPDZ	24	70	**20**	40	15	⊥
	MPyC	**9**	⊥	⊥	700	⊥	⊥
	Obliv-C[3]	140	⊥	⊥	⊥	⊥	100
	Oblivm	44	⊥	⊥	⊥	⊥	1,500
	Picco[2]	19	N/B	N/B	N/B	N/B	⊥
	Sharemind	15	⊥	⊥	⊥	2,500	⊥
	Sequre	**9**	**50**	95	–	–	⊥

[a] GC: garbled circuits.
[b] Obsolete, or unable to run.
[c] Unlike other solutions that employ brute-force strategy, Obliv-C uses a dynamic programming approach.

as *study participants*), we will here assume without the loss of generality to have only three computing parties (CP_0, CP_1, and CP_2), or to be more precise, two standard computing parties (CP_1, CP_2) and an auxiliary computing party (CP_0; also known as *trusted dealer*). We will denote each party's portions of the data with the angled brackets: $\langle a, b, c, d \rangle$ means that a, b, c, and d are accessible by CP_1, CP_2, CP_0, and SP, respectively. We will adhere to a convention that $\langle a, b \rangle \equiv \langle a, b, \perp, \perp \rangle$ and $\langle a, b, c \rangle \equiv \langle a, b, c, \perp \rangle$, where $\perp$ denotes an empty slot. Further, a pair of secret shares $\langle x_1, x_2 \rangle$ of x will be often shortened as $[x]$. Also, unless explicitly noted, each line of pseudocode is executed on all computing parties in parallel. Lastly, unless otherwise noted, each routine operates on top of an algebraic structure S, which can interchangeably be the Galois field $\mathbb{Z}_p$ or a finite $\mathbb{Z}_{2^k}$ ring.

Table S6 Runtime and network stats for secure GWAS, secure DTI, secure Opal, and secure Ganon. Sequre is presented with and without the compile-time optimizations enabled (i.e. network, pattern-matching, matrix and modulus optimizations). Note that we were unable to set-up and run SyMPC/PySyft on a LAN network. Runtime is given in hh:mm:ss format. Online bandwidth is expressed in MB. Beaver partitions are expressed in millions.

		Runtime	Bandwidth	Beaver partitions
GWAS	C/C++	2:43:10	10,254	2.41
	Sequre (w/o opt)	6:46:46	151,309	3.61
	Sequre (w/ opt)	0:48:40	9,886	2.34
DTI	C/C++	0:10:16	683	1.04
	SyMPC (w/o opt)	N/A	N/A	N/A
	Sequre (w/o opt)	0:13:17	567	1.04
	Sequre (w/ opt)	0:06:08	510	1.04
Opal	Sequre (w/o opt)	19:26:40	55,035	402.21
	Sequre (w/ opt)	15:54:22	33,941	290.49
Gan.	Sequre (w/o opt)	44:39:08	96,141	55.88
	Sequre (w/ opt)	20:30:52	81,186	55.72

4.2 Sequre's standard library

This section provides an overview of the MPC routines included in Sequre's standard library. Most of the procedures are adopted from external sources such as Cho et al. [9]; for more details, please consult the appropriate literature and the accompanying supplementary materials. For procedures that have been newly developed for Sequre, like our variant of linear classifier, an MPC pseudocode of the procedure together with the Sequre code listing is provided below.

Sequre's standard library includes 24 algebraic protocols from Cho et al. [9]: secret sharing and revealing routines, private and public addition and multiplication, private fixed-point arithmetic, private division and square root calculation, private exponentiation and polynomial evaluation, private and public bitwise operators, private and public comparison operators, and private linear algebra routines (householder transformation, QR decomposition, tridiagonalization, eigendecomposition and orthonormal basis calculation). It also includes naïve oblivious array getter [36] and oblivious dictionary with private indexing, private bit decomposition and bitwise addition [87]), and private linear support vector machine.

The implementation details for all procedures can be found in the referenced sources. However, because the core concepts, such as basic secure arithmetic, are necessary for understanding Sequre's optimization procedures later on, we will provide a short overview below.

4.2.1 Revealing

The exact procedure for secretly sharing private data between the multiple computing nodes is described in Section 2. The procedure for revealing the secretly shared value in additive secret-sharing schemes follows the opposite steps: a value is revealed by adding up the secretly shared values together. In other words, $x = [x]_1 + [x]_2$.

4.2.2 Addition

Adding the two numbers in additive secret-sharing-based schemes is straightforward procedure. Namely, for the sum of two secret-shared numbers $[x]$ and $[y]$ we just add the respective shares together: $[x+y] = \langle [x]_1 + [y]_1, [x]_2 + [y]_2 \rangle$. Note that revealing

the obtained shares reveals the value of $x + y$. However, when adding a public value a to a secret-shared number it suffices to add it to one and only one of the shares: $[x] + a = \langle [x]_1 + a, [x]_2 \rangle$. For detailed MPC pseudocodes for the procedures above, see protocols 3 and 4 in Cho et al. [9].

4.2.3 Multiplication and Beaver partitions

Multiplying a secret-shared number $[x]$ with a public number a is straightforward in an additive secret sharing scheme. Namely, to compute the shares of such product, it suffices to multiply each share of x with the public number: $[ax] = \langle a[x]_1, a[x]_2 \rangle$.

On the other hand, multiplying the two secret-shared values is not as easy. Note that if the shares at each computing party are simply multiplied together we obtain incorrect result: $[x]_1[y]_1 + [x]_2[y]_2 \neq xy$. This problem can be avoided by utilization of the *Beaver multiplication triplets* [57]. In this method, in order to compute the shares of a product $[xy]$ we first sample and secretly share the three random numbers $a \in S$, $b \in S$, and $c = ab \in S$ and then reveal the value of $x - a$ and $y - b$ to finally compute $\langle (x-a)(y-b) + (x-a)[b] + (y-b)[a] + [c] \rangle$. Revealing the last value yields $(x - a)(y - b) + (x - a)b + (y - b)a + ab$ which is equal to xy.

Sequre, however, implements an improved variant of Beaver triplets multiplication that introduces a concept of *Beaver partitions* and leverages a trusted dealer CP_0 and the streams of pseudo-random generators to reduce the online communication between the computing parties. Specifically, Beaver partitions of a secret-shared number $[x]$ are defined as the values $\langle x - r_x, x - r_x \rangle$ and $\langle [r_x]_1, [r_x]_2, r_x \rangle$, where $r_x \in S$ is randomly sampled by a trusted dealer and secretly shared to the computing parties. To multiply the two secret-shared numbers $[x]$ and $[y]$, it suffices to multiply their Beaver partitions in a cross-over fashion: $[xy] = (x - r_x)[r_y] + (y - r_y)[r_x] + [r_x r_y] + (x-r_x)(y-r_y)$, where $(x-r_x)(y-r_y)$ is added publicly and $r_x r_y$ is computed at CP_0 and secretly shared to other computing parties. For details, see protocols 6 and 7, as well as supplementary notes 3 and 7, in Cho et al. [9].

4.2.4 Generalized polynomial evaluation

Sequre implements a generalized polynomial evaluation routine [9] for evaluating the generalized polynomial form

$$P_m(x_1, x_2, \ldots, x_n) = \sum_{i=1}^{m} c_i \prod_{j=1}^{n} x_j^{p_{ij}} \ , \ c_i, x_j, p_{ij} \in \mathbb{R}$$

with the minimal number of the online communication rounds. For evaluating this polynomial, it suffices to compute the Beaver partitions $x_j - r_j, r_j$ for each variable x_j only once and then compute the expansion of the polynomial terms $x_j^{p_{ij}} = ((x_j - r_j) + r_j)^{p_{ij}}$ offline—within each computing party—in order to evaluate the polynomial. However, the expansion of the offline terms is exponential with respect to the polynomial degree and can be impractical if the polynomial degree is too high. In this case, a simple series of traditional multiplication routines often performs better.

Sequre supports a generalized polynomial approach but hides it from the end-user. It is only utilized within the network optimizations (Section 4.3.2) where the infeasible expansion of the terms can be detected and avoided at a compile-time.

4.2.5 Oblivious data structures

Data structures with secret indexing—also known as *oblivious data structures*—enable secret indexing of the secret data, where both the data and the indices by which the data is accessed are secretly shared. For example, the traditional secret-shared array has its values shared between the computing parties, but the indexing of the array is still public (i.e. we can still access the i-th element of a secretly shared array $[x]$ as $[x][i]$ at each computing party). In an oblivious array, the index i is also secretly shared, so there is a need to allow array access via the $[i]$ at each party (i.e. $[x][[i]]$). Sequre implements a naïve oblivious array getter [36]. Additionally, it supports a private dictionary with secret indexing, where both the keys and values of the dictionary can be private. It also extends a table lookup routine from Cho et al. [9] to implement a secure getter for a private dictionary.

Algorithm 1 Oblivious dictionary construction

INPUT: $D = \{k_i \rightarrow v_i\}_1^n$: a public dictionary given as a key-value mapping

OUTPUT: $[D] \in S^n$: a private dictionary represented as a private array of Lagrange coefficients

1: $c_1, c_2, \ldots, c_n \leftarrow \text{LagrangeInterpolation}(D)$
2: $[D] \leftarrow \text{SecretShare}(c_1, c_2, \ldots, c_n)$

Algorithm 2 Oblivious dictionary getter

INPUT: $[D] \in S^n$: a private dictionary represented as a private array of Lagrange coefficients

$[k] \in S$: a Secret-shared key to query the dictionary with

OUTPUT: $[v] \in S$: a secret-shared value that corresponds to k in D

1: $[k^2], \ldots, [k^{n-1}] \leftarrow \text{Powers}([k], n-1)$
2: $[v] \leftarrow [D] \cdot (1, [k], [k^2], \ldots, [k^{n-1}])$

Note that this approach makes the implementation of the dictionary setter difficult, because we have to re-construct the dictionary whenever a new key-value pair is added to it.

4.2.6 Linear support vector machines (SVM)

The linear SVM from Section 1 is provided in Sequre's standard library. As mentioned there, it is a binary classification algorithm that uses stochastic gradient descent to minimize the regularized Hinge loss. To be more specific, we minimize the loss $l \in \mathbb{R}$ for $L : \mathbb{R}^n \rightarrow \mathbb{R}$ such that

$$l = L(\mathbf{w}, b) = \lambda \|\mathbf{w}\|^2 + max(0, 1 - t(\mathbf{w}^T \cdot \mathbf{x} - b)))$$

where $\mathbf{w} \in \mathbf{R}^n$, $\mathbf{x} \in \mathbf{R}^n$, $b \in \mathbf{R}$, and and $t \in \{0, 1\}$ are respectively the regression weights vector, input feature vector, the bias, and the truth value.

We need to minimize l with respect to $\mathbf{w}$ and b via stochastic gradient descent. Thus we need to iteratively translate $\mathbf{w}$ and b by a predefined step size $\eta \in \mathbb{R}$ in the negative direction of a gradient of $L(\mathbf{w}, b)$ as follows:

$$\mathbf{w}_{k+1} = \mathbf{w}_k - \eta \nabla_{\mathbf{w}_k} L(\mathbf{w}_k, b)$$

$$b_{k+1} = b_k - \eta \nabla_{b_k} L(\mathbf{w}_k, b)$$

where $\mathbf{w}_0$ and b_0 can be picked at random. Note that

$$\nabla_{\mathbf{w}} L(\mathbf{w}, b) = 2\lambda \mathbf{w} - t \cdot \left[(1 - t(\mathbf{w}^T \cdot \mathbf{x} - b)) > 0 \right] \cdot \mathbf{x}$$

$$\nabla_b L(\mathbf{w}, b) = t \cdot \left[(1 - t(\mathbf{w}^T \cdot \mathbf{x} - b)) > 0 \right].$$

The pseudocode for the described procedure is provided in Algorithm 3. The secure MPC variant of the same algorithm is provided in Algorithm 4.

Algorithm 3 Linear SVM training

INPUT:

features $\in \mathbb{R}^{m \times n}$: features matrix

labels $\in \mathbb{R}^m$: list of labels

eta(η) $\in \mathbb{R}$: step size

epochs $\in \mathbb{Z}$: number of epochs

lambda(λ) $\in \mathbb{R}$: L_2 regularization factor

OUTPUT:

$w \in \mathbb{R}^n$ and $b \in \mathbb{R}$: a weights vector and a bias that minimize the Hinge loss of the classifier output

```
1: w ← (1, 1, . . . 1) ∈ ℝ^n
2: b ← 1
3: for i ∈ \overline{0, epochs} do
4:     for x ∈ features ∧ t ∈ labels do
5:         y ← w · x − b
6:         w ← w · (1 − 2λη) + η · t · ((1 − t · y) > 0) · x
7:         b ← b − η · t · ((1 − t · y) > 0)
8:     end for
9: end for
```

The Sequre source code does not differ much from Algorithm 3:

Code 10 Linear SVM training in Sequre

```
from sequre import dot, zeros_like

mpc, (features, labels) = pool_shares()

@sequre
def lsvm(mpc, X, Y, eta, epochs, l2):
  w = zeros_like(X[0]) + 1
  b = zeros_like(Y[0]) + 1

  for i in range(epochs):
    for feature_vector, label in zip(X, Y):
      z = dot(mpc, feature_vector, w) - b
      # Backward pass
      grad_b = label * ((1 - z * label) > 0)
      grad_w = w * l2 * 2 - feature_vector * grad_b
      w = w - grad_w * eta
      b = b - grad_b * eta

  return w, b
```

The inference can be done in a single forward pass as follows:

Algorithm 4 Linear SVM training in MPC

INPUT:

[features] $\in \mathbb{Z}_p^{m \times n}$ - secret shared features matrix

[labels] $\in \mathbb{Z}_p^m$ - secret shared list of labels

eta $\in \mathbb{Z}_p$ - public step size

epochs $\in \mathbb{Z}$ - public number of epochs

lambda $\in \mathbb{Z}_p$ - L_2 regularization factor

OUTPUT:

[w] $\in \mathbb{Z}_p^n$ and [b] $\in \mathbb{Z}_p$ - secret shared weights vector and a bias that minimize the Hinge loss of the classifier output

```
[w] ← secret_share((1, 1, ..., 1))
[b] ← secret_share(1)
for i ∈ ‾0, epochs‾ do
    for [x] ∈ [features] ∧ [t] ∈ [labels] do
        [y] ← beaver_dot_product([w], [x])
        [y] ← truncate([y]) − b
        [c] ← η · [t]
        [c] ← truncate([c])
        [c1] ← (1 − y)
        [s] ← is_positive(c1)
        [c] ← [c] · [s]
        [v] ← beaver_multiply([x], [c])
        [v] ← truncate([v])
        [w] ← [w] · (1 − 2λη) + [v]
        [b] ← [b] − [c]
    end for
end for
```

Code 11 Linear SVM inference in Sequre

```python
from sequre import dot

@sequre
def lsvm_predict(mpc, x, w, b):
  return dot(mpc, x, w) - b
```

4.2.7 Shared tensor and supported operations

Sequre operates on top of tensors of arbitrary dimension. The SharedTensor class that implements them stores the secret additive share and auxiliary data, such as Beaver partitions, as n-dimensional arrays at each computing party. Furthermore, the compile-time optimizations apply only to SharedTensor expressions. Table S7 presents all secure operations supported for SharedTensor operands.

Additionally, secure protocols that are enabled on top of arbitrary n-dimensional arrays but not yet added to the SharedTensor are presented in Table S8. Note that calling these protocols manually on top of a SharedTensor is seamless as they can be called directly on top of the share attribute of the class.

4.3 Compiler optimizations

4.3.1 Beaver caching optimization

As mentioned in Section 4.2.3, our variant of secure multiplication $[x][y]$ necessitates obtaining Beaver partitions $(x - r_1, [r_1])$ and $(y - r_2, [r_2])$ beforehand. Note that the parts of Beaver partitions are known to all computing parties, while the actual

Table S7 Supported SharedTesor operations in Sequre. x and y are shared tensors. Some operations are supported only for 2-dimensional (matrices) or 1-dimensional (vectors) shared tensors.

Secure operation	Example usage
Element-wise addition / subtraction	x + y; x - y
Element-wise multiplication	x * y
Element-wise exponentiation	x ** c (c is a constant)
Element-wise division	x / y
Element-wise comparisons	x == y; x != y; x > y; x < y; x >= y; x <= y
Element-wise equare root	sqrt(x)
Dot product / Matrix multiplication	dot(x, y); matmul(x, y)
Element-wise absolute value	abs(x)
Max/min element (vector only)	max(x); min(x)
Argmax/argmin element (vector only)	argmax(x); argmin(x)
Householder transformation (matrix only)	householder(x)
QR factorization (matrix only)	qr_fact_square(x)
Tridiagonalization (matrix only)	tridiag(x)
Eigen decomposition (matrix only)	eigen_decomp(x)
Orthonormal basis (matrix only)	orthonormal_basis(x)
Element-wise bit decomposition	bit_decomposition(x, . . .)
Element-wise bit-wise addition	bit_add(x, y)

Table S8 Additional Sequre operations supported on top of raw n-dimensional arrays but not the shared tensors.

Secure operation	Method path
Oblivious getter	sequre.collections.oblivious_get
Polynomial evaluation	sequre.polynomial.evaluate_poly

values of x and y remain hidden as $[r_1] \in S$ and $[r_2] \in S$ are randomly generated and secret-shared between the parties.

It suffices to compute the Beaver partitions only once for each unique secret share (i.e., a variable) and intermediate product within an arithmetic expression—regardless of the number of the occurrences of each variable—and then reuse the partitions in the subsequent multiplications. This simple approach yields a non-marginal network performance improvement [9]. However, the optimal reuse strategy requires MPC designers need to carefully inspect the code and manually partition each variable before reusing the partitions throughout the codebase. The manual inspection often fails to uncover every reuse opportunity within the codebase due to the cumbersome and convoluted nature of MPC routines. Sequre automates this process by statically analyzing the binary expression tree of each arithmetic expression within each independent code block, and by extracting all multiplication operators from these expression. The variables within expressions are partitioned upstream immediately after their instantiating. Each partitioned variable will then carry its partitions throughout expressions during the runtime, and will either propagate them to the result in case it is used for addition, or utilize them in case of multiplication. Partitions will be invalidated if a new value is assigned to the partitioned variable (e.g., `a = 5` will invalidate all partitions of the variable `a`), and the variable will be re-partitioned if it is utilized as a multiplicative factor in the subsequent expressions. Note that a similar functionality could have been achieved by just partitioning and caching each variable's partition immediately before each multiplication. However, such simple strategy would fail to optimize the following edge case:

Code 12 Beaver caching edge case

```
...
a = b * c
# a should be immediately partitioned here
d = a + 1
e = a + 2
# Partitioning e and d here could have been avoided
# if a was partitioned immediately after it was instantiated above
f = e * d
...
x = m * n
# However, x should not be partitioned here as it would be unnecessary
return x
```

Constructing the binary expression tree and its subsequent static compile-time analysis is implemented a Seq's Intermediate Representation (IR) [47] pass in over 500 lines of code. The propagation and utilization of partitions during runtime is implemented directly in Seq.

4.3.2 Polynomial optimization

Generalized polynomial evaluation (Section 4.2.4) securely evaluates the polynomials of the form

$$P_m(x_1, x_2, \ldots, x_n) = \sum_{i=1}^{m} c_i \prod_{j=1}^{n} x_j^{p_{ij}},\ c_i, x_j, p_{ij} \in \mathbb{R} \tag{1}$$

with the minimal network overhead. This procedure only calculates the Beaver partitions of the polynomial variables by partitioning each variable only once per polynomial evaluation. No intermediate product requires further partitioning. The polynomial terms are expanded and computed offline, and as such require no additional communication between the computing parties.

To capitalize on this, Sequre will track and transform the series of arithmetic expressions in each code block into their polynomial form and then utilize the polynomial evaluation routine from the standard library to evaluate them. Akin to the Beaver caching analysis, Sequre constructs a binary expression tree on top of a series of expressions in a block and recursively expands its multiplication and exponentiation nodes (Figure S3) to reach the form of generic polynomial in (1). The polynomial coefficients and exponents are then parsed from the expanded binary expression tree and passed to the polynomial evaluation method.

Binary expression tree analysis, transformation, parsing, and scaffolding for the polynomial evaluation method are done at the compile-time and implemented as a Seq IR pass [47] in over 800 lines of C/C++ code. Secure polynomial evaluation, as a part of Sequre's standard library, is then executed at runtime.

While this optimization minimizes the number of Beaver partitions and reduces the bandwidth to a theoretical minimum, the expansion of the polynomial terms can still be expensive. Hence, for the expressions that produce large polynomial degrees, the overall approach will be infeasible both at compile-time (due to the expansion of the terms) and runtime (where another series of terms expansion that stems from Beaver partitions is required; Supplementary Note 3 in Cho et al. [9]). To avoid this problem, Sequre will calculate the size and the degree of the eventual polynomial

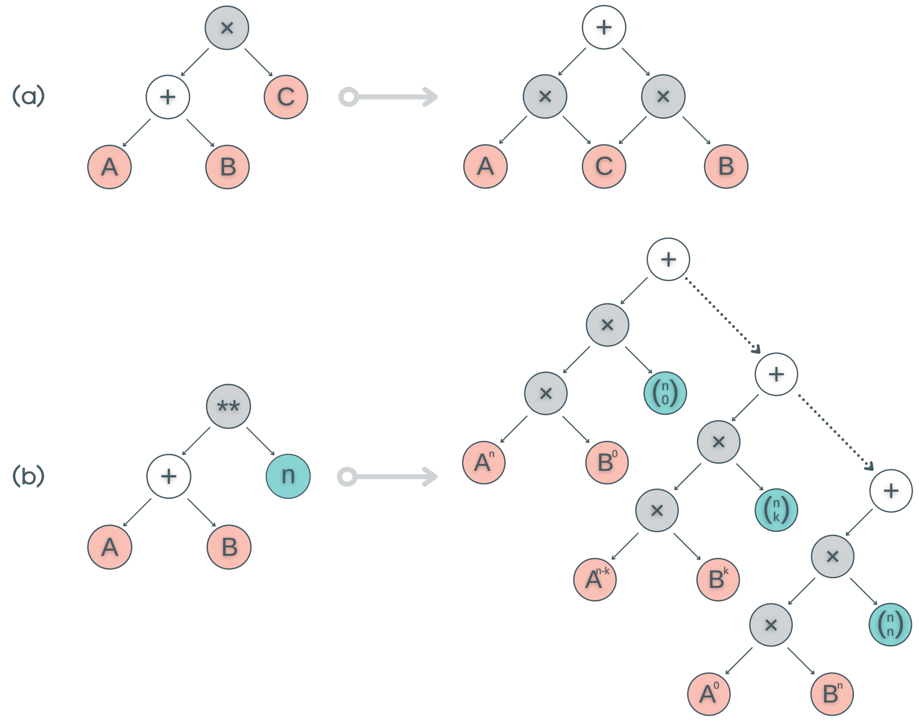

Fig S3 Binary expression tree term expansion for (a) multiplication and (b) exponentiation. The two transformations will be applied to the nodes recursively until the structure of the tree reaches the shape of generalized polynomial (1).

first and then decide whether to proceed with the polynomial optimization or the Beaver caching optimization above. It is important to note that the polynomial optimization is not just another variant of Beaver caching optimization—instead, these two optimizations are mutually exclusive. Currently, the choice between the two optimizations is decided by the predefined threshold for the degree of the polynomial. This threshold is currently hardcoded to 5 and was experimentally chosen based on observing the network latency and runtimes under different values.

4.3.3 Pattern matching optimization

The specific nature of MPC operations allows ample opportunities for algebraic operation optimizations, such as division and square root optimizations. For example, calculating a square root in an MPC environment produces its multiplicative inverse as a by-product. So instead of invoking the two expensive protocols (division and square root) to securely compute ${}^{[a]}/\sqrt{[b]}$ where $[a], [b] \in S$ are secret shares of $a \in \mathbb{R}$ and $b \in \mathbb{R}$, it is more efficient to multiply the dividend by the multiplicative inverse of the square root (i.e., $[a] \cdot \sqrt{[b]}^{-1}$). Similarly, division by a public number can be replaced by multiplication (see Code 4.3.3). Sequre implements an intermediate representation pass that identifies such patterns and replaces them with the more efficient counterparts.

Code 13 Pattern-matching transformations

```
a = b / sqrt(c)
# Transformed to:
... a = b * sqrt_inverse(c)

a = fixed_point_share / 3.4
# Transformed to:
... a = fixed_point_share * to_fixed_point(1 / 3.4)
... a = truncate(a)

a = non_fixed_point_share * 3.4
# Transformed to:
... a = to_fixed_point(non_fixed_point_number) * to_fixed_point(3.4)
... # There is no need for truncation

a = fixed_point_share * 3
# No transformations needed.
```

Operating on top of the finite fields or rings allows only integer arithmetic. For that reason, non-integers are usually converted to a fixed-point [88] or a floating-point [89] representation. Conversion between the integer and non-integer types is usually manually implemented in today's secure MPC protocols. For example, multiplying two fixed-point numbers mandates utilizing a secure truncation protocol to cut the fractional part in half. If, however, one of the factors is not a fixed-point number, then the truncation must not be employed. Sequre uses the fixed-point representation of non-integers, and defaults to 20 bits reserved for the fractional part, 40 bits for the whole fixed-point numebr, and 60 bits for the statistical security. Also, Sequre handles the casting between the types and the automatic truncation of products implicitly as it is done in general-purpose languages such as C or C++.

4.3.4 Matrix arithmetic optimizations

If all matrix operands are diagonal during the element-wise matrix operations, Sequre provides a special class `SharedTensor` that can optimize these operations by computing the values only over the diagonals.

Sequre also employs a vectorized Strassen algorithm [90] for multiplying large matrices when the size of a matrix exceeds 250,000 elements. Strassen's algorithm is a recursive divide-and-conquer paradigm and, in Sequre, the matrix products in the leaves of the recursion are multiplied via LLVM's vectorized matrix multiplication instruction. For matrices that contain fewer elements, a straightforward matrix multiplication algorithm is employed, with the latter operand being transposed to maximize the utilization of lower levels of CPU cache.

4.3.5 Multiple algebraic structures

Enabling the MPC protocols to operate on top of $\mathbb{Z}_{2^k}$ rings instead of Galois fields is a common practice today because the rings are generally faster on modern computer architectures [56]. Sequre supports running most of its standard library routines in both algebraic structures (see Figure S4).

However, some MPC protocols such as fixed-point value comparisons, division, and square root calculation—all operating on top of the fixed-point values—exclusively work with finite Galois fields in Sequre. Hence, when operating on top of a $\mathbb{Z}_{2^k}$ ring, Sequre needs to internally switch to a finite Galois field when calling such procedures. This switch is not free, because the difference between the sizes of the two algebraic structures is publicly subtracted from the input and then publicly

Generalized beaver triplets	On field (Z_p)	On ring (Z_{2^k})
Multiplication	✓	✓
Exponentiation	✓	✓
Polynomial evaluation	✓	✓
Table lookup	✓	✗

Fixed-point arithmetic	On field (Z_p)	On ring (Z_{2^k})
Truncation	✓	✓
Division	✓	⇄
Square root	✓	⇄

Oblivious data structures	On field (Z_p)	On ring (Z_{2^k})
Oblivious array	✓	✓
Oblivious dictionary	✓	✗

"Boolean's"	On field (Z_p)	On ring (Z_{2^k})
Bitwise operations	✓	✗
Comparison	✓	⇄

Linear algebra	On field (Z_p)	On ring (Z_{2^k})
Householder	✓	✓
QR factorization	✓	✓
Tridiagonalization	✓	✓
Eigen decomposition	✓	✓
Orthonormal basis	✓	✓

✓ - Fully supported
✗ - Not supported
⇄ - Supported by resorting to fields

Fig S4 Finite Galois fields and $\mathbb{Z}_{2^k}$ rings support in Sequre.

added to the output of each procedure. If the sum of secret shares does not overflow the predefined size of the algebraic structure, the switch will change the accuracy of the procedure. The change in accuracy is equal to $\tau = (p - r)/2^f$, where p, r, and f are the size of a field, ring and the fractional portion of the fixed-point number, respectively. Sequre's default fixed-point arithmetic setup evaluates this value to $\tau = 1/2^{20}$. Note that the comparison between the two fixed point values a and b will yield incorrect results on $\mathbb{Z}_{2^k}$ rings if the difference between a and b is within the $(0, \tau]$ half-range. Finally, the size of f is configurable in Sequre, which can coerce τ to be arbitrarily small and hence minimize the practical chance of the error. Hence, setting the size of the finite field to be as similar as possible to the size of the ring, and increasing the fractional part of the fixed-point numbers—something that Sequre does by default—is needed for the improved accuracy (note that the sizes of the field and the ring must differ since the only case where Z_p is equivalent to Z_{2^k} is when $p = 2^k = 2$—a value too impractical for arithmetic-circuits based MPC setups). Note that the procedures that are sensitive to any errors (e.g., table lookup that underpins the Sequre's implementation of oblivious dictionaries, or bitwise operations) operate only on finite fields in Sequre.

5 Benchmark and hardware setup

For benchmarking secure GWAS, secure DTI inference, and secure metagenomic binning, we used the test-case scenarios and benchmark configurations from [9], [10], and [23] respectively. For comparing Sequre against other frameworks, we adopted benchmarks from [21]: `mult3` for a series of secure multiplications, `innerprod` for inner product between two private vectors, and `xtabs` for a cross-tabular aggregation of oblivious arrays. The first two benchmarks were slightly adapted for scalability by extending a series of multiplications into a series of additions and multiplication and by setting the vectors' length in the second benchmark to be $100,000$ instead of the original 10.

For the sake of precise measurements, all benchmarks were executed on a single machine where each party (computing or collaborating node) was run as a separate

process. Network communication was established through inter-process communication sockets (`AF_UNIX`). Additionally, GWAS, DTI, Opal, and Ganon where evaluated on a local-area network with a different machine for each computing party. Single-machine results for GWAS were evaluated on Intel® Xeon® Platinum 8260 CPU at 2.40 GHz with 192 logical cores and 1 TB of RAM. All the other benchmarks, including the local-area network runs, were evaluated on multiple Intel® Core™ i7-8700 CPU at 3.20 GHz with 12 logical cores and 60 GB of RAM.

Table S9 List of tools, versions, and datasets used in each application (Secure-GWAS, Secure-DTI, Ganon, Opal) and MPC frameworks benchmarked against Sequre. GWAS lung cancer dataset was sampled into first 3,000 individuals and 30,000 SNPs. For tools that do not use a versioning scheme, the shortened commit hash of the version used is included.

	Tool	Version	Dataset
GWAS	Clang	14.0.0	Lung cancer dataset (accession: phs000716.v1.p1)
	GMP	6.2.1	
	NTL	10.3.0	
DTI	Clang	14.0.0	Reduced STITCH dataset: `https://bit.ly/3AuhaPn`
	GMP	6.2.1	
	NTL	10.3.0	
	Python	3.8.11	
	Syft	0.5.3	
	SyMPC	0.5.0	
Opal	Python	3.6.13	Opal dataset: `http://giant.csail.mit.edu/opal/data.tar.bz2`
	VowpalWabbit	8.11.0	
Ganon	Seq	0.10.1	Opal dataset: `http://giant.csail.mit.edu/opal/data.tar.bz2`
	Clang	14.0.0	
	SeqAn	3.1.0	
MPC frameworks	ABY	08baa85	N/A
	EMP	0.2.3	
	Frigate	4ef001b	
	Jiff	8ea565d	
	MP-SPDZ	0.1.5	
	MPyC	0.8	
	SyMPC	0.5.0	
	Obliv-C	2bacf04	
	Oblivm	50ed0fb	
	Picco	ee85c91	
	Sharemind	2017.12	
	Sequre	0.0.1	

Lastly, Table S9 enlists all the tools, versions and the links to datasets used for benchmarks.
